# Waste jean derived self N-containing activated carbon as a potential electrode material for supercapacitors

**DOI:** 10.55730/1300-0527.3579

**Published:** 2023-07-08

**Authors:** Yavuz GÖKÇE

**Affiliations:** Department of Chemical Engineering, Faculty of Engineering, Ankara University, Ankara, Turkiye

**Keywords:** Waste textile product, activated carbon, energy storage, supercapacitor

## Abstract

The rapid rise of the world population increases the annual amount of waste textile products. Textile products create a significant amount of CO_2_, water, and chemical footprints during production. Therefore, the reusability of textile products has an important environmental and economic impact. Waste denim was used in this study to produce activated carbon (AC) samples as the alternative substance for supercapacitor electrodes. Characterisation studies showed that AC samples contain nitrogen originating from the elastane in the denim structure. Electrochemical characterisation tests proved the pseudocapacitive behaviour of the denim-derived AC due to the nitrogen content. Specific capacitance values observed for the three-electrode and two-electrode cell configurations were 95.93 F/g and 54.64 F/g at 1 A/g, respectively. Good capacitive retention (83.01%) of the cell after 3000 galvanostatic charge-discharge cycles at 1 A/g shows that waste denim can be considered as raw material for energy storage systems.

## 1. Introduction

The world population growth after the industrial revolution has caused many problems [1,2]. In the last century, the increase in world population and technological developments have increased the dependency on energy [3]. Until recently, most of the energy needed was provided by fossil fuels. The adverse environmental impacts caused by using fossil fuels have led to research on new energy sources and more efficient use of energy [4]. The continuity in energy needs has enabled the development of energy storage systems that convert energy into different forms as soon as it is produced and used in case of need. In particular, the spread of portable electronic devices and new vehicle technologies increased the number of studies on electrochemical energy storage systems [5–7]. Electrode materials are the most important component in electrochemical energy storage systems. Energy storage performance varies according to the electrode materials type, composition, and structure. There are many studies on producing different electrode materials and their use in energy storage studies [8–13]. Electrode materials in the electrochemical energy storage systems are often produced from carbon-based materials. The most preferred carbon-based materials in commercial electrode production are activated carbons (AC). AC can be obtained from several carbonaceous materials, such as biomasses [14], polymers [15], coals [16], and waste materials [17].

The abundance, cheapness, processability, and easy accessibility of the electrode material are important factors affecting the choice of raw material. Among waste materials, textile wastes are quite remarkable. Textile wastes have a global status, and their amount is increasing every day. Annual fibre production, which currently exceeds 110 million t, is estimated to increase to 146 million t in 2030 [18]. Most textile wastes are transferred to landfills or municipal combustion plants and only a limited percentage have been recovered [19]. Instead of destroying textile wastes in incineration plants, converting them into activated carbons as a value-added material is an alternative recycling practice. Since a new application area will be created by using textile wastes, the footprints of carbon, water, and solid waste caused by textile products will be reduced to a certain extent. Besides, the cost of energy storage devices will be reduced by using waste textile-derived AC electrodes. There are some published studies in the literature related to the conversion of textile product wastes to AC [20–26]. However, a limited number of these studies aimed to use textile waste-derived AC for supercapacitor applications [27–31].

The purpose of the study is to prepare the AC from the waste denim that cannot be reused as a textile product and to use the AC product as electrode material in the supercapacitor cells. It is also aimed to take advantage of the pseudocapacitive contribution of the produced activated carbon by using a self-nitrogen-containing textile waste.

## 2. Materials and method

### 2.1. Materials

Waste jean with 68% cotton, 30% polyester, and 2% of elastane composition was used as the raw material to produce AC. Potassium hydroxide (KOH, Merck) was the activating chemical. 1 M sulfuric acid (H_2_SO_4_, Riedel-de-Haen) was employed as aqueous electrolyte. Conductive carbon black (CB, TIMCAL Super C45), polyvinylidene fluoride (PVDF, Sigma-Aldrich), and n-methyl-2-pyrrolidone (NMP, Acros Organics) were the chemicals used to assemble supercapacitor electrodes.

### 2.2. Production of activated carbon

**Figure 1:**
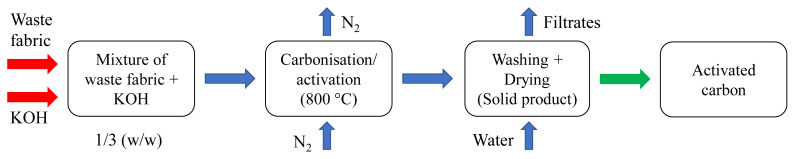
Production of AC800 samples.

The waste jean was first dried at 105 °C for 2 h before activation. Dried raw material (1 g) was cut into small pieces and immersed into 20 mL of KOH solution. The weight ratio between raw material and KOH was 1:3. The slurry was kept at room temperature for 2 h to thoroughly wet the fabric samples and then placed into the oven to remove the moisture at 105 °C for 12 h. Then, it was poured into the ceramic crucible and put into a muffle furnace. The solid mixture was carbonised and activated at 800 °C with a heating rate of 10 °C/min. The total carbonisation/activation time was 1.5 h. After the carbonisation/activation process, the solid residue was filtered and washed using hot distilled water to remove excess KOH until pH 7. The AC samples were placed in an oven to obtain dry AC samples (labelled as AC800) at 105 °C for 12 h for further characterisation and electrochemical studies. Figure 1 shows the production steps of AC800.

### 2.3. Electrode preparation and electrochemical characterisation

A mixture of AC (85%), CB (10%), and PVDF (5%) was used to prepare the electrodes for the electrochemical performance tests. Appropriate amounts of AC and CB were weighed and mixed in a beaker. Then, the solid mixture was poured over PVDF solution (NMP: solvent). The slurry was stirred in a closed vessel for 30 min and ultrasonicated for 10 min to obtain a more homogenous mixture. The electrodes were assembled by spraying the ink onto the stainless-steel current collectors.

The electrochemical performances of the AC800 were tested by using three-electrode and symmetric two-electrode configurations. The dimensions of the current collectors used for the two-electrode and three-electrode configurations were 1 cm × 1 cm and 0.8 cm × 1.25 cm, respectively. The electrodes were vacuum dried to evaporate NMP at 80 °C for 24 h.

Electrochemical analyses in the presence of 1 M H_2_SO_4_ aqueous electrolyte were tested with using Gamry Reference 3000 potentiostat. Ag/AgCl (in 3 M KCl) was the reference electrode, and a platinum wire (0.406 mm diameter, immersed length: at least 2.5 cm) was used as an auxiliary electrode for the three-electrode cell configuration. Cyclic Voltammetry (CV) analyses were conducted in the potential window of 0 V–0.8 V at different scan rates between 2–200 mV/s. GCD analysis was also carried out for the long-term performance tests. Electrochemical Impedance Spectroscopy (EIS) tests were completed in a frequency range from 10 kHz to 0.01 Hz with an amplitude of 5 mV (rms). Specific capacitance values of AC800 were determined by Galvanostatic Charge-Discharge (GCD) analysis between 0 V–0.8 V at different current densities (0.25–20 A/g) for the three-electrode and two-electrode cell configurations according to Equation 1 (Cs (F/g, specific capacitance of the cell), I (A, applied current), Δt (s, duration of discharge step), m (g, mass of the total active material on the electrode) and ΔV (V, change in potential difference for the discharge process after the IR drop)).


(1) $$C_{s}=\frac{I\Delta t}{m\Delta V}$$

Energy densities (Equation 2) and power densities (Equation 3) of AC800 were calculated as follows:


(2) $$E=\frac{C_{s}{\left(\Delta V\right)}^{2}}{2x3.6}$$

(3) $$P=3600\frac{E}{\Delta t}$$

where E (Wh/kg) is energy density and P (W/kg) is power density.

### 2.4. Characterisation

The textural properties of AC800 were characterised by using a Quantachrome NOVA 2200 analyser. The surface area (S_BET_) and the pore size distribution (PSD) values were determined according to Brunauer-Emmett-Teller (BET) and Non-local Density Functional Theory (NLDFT) methods, respectively. Surface functionalities on AC products were identified with a Fourier Transform Infrared (FTIR) spectrophotometer (Shimadzu FTIR-8040) by using AC800-KBr pellet at a weight ratio of 1: 1400. The pellet was dried to avoid moisture in the sample. The spectrum was taken between 4000–400 cm^−1^ wavenumber range. Surface characterisation of AC800 in terms of surface functionalities was conducted by X-ray photoelectron spectroscopy (XPS, Thermo NEXSA XPS) equipped with a monochromated Al Kα X-ray source (1486.7 eV).

## 3. Results and discussion

### 3.1 Textural properties

**Figure 2:**
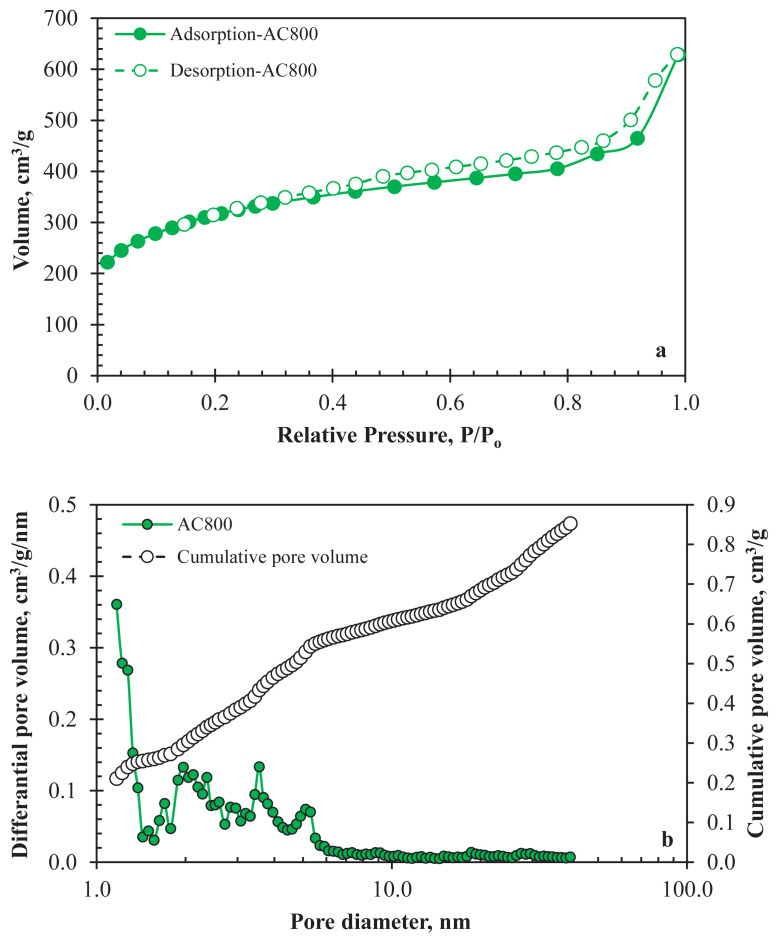
a) N_2_ adsorption-desorption isotherms, b) PSD and cumulative pore volume of AC800.

N_2_ adsorption-desorption isotherms of AC800 are shown in Figure 2a. The sharp increase in the adsorption isotherm indicates the sample microporosity. A hysteresis loop can be seen in the figure and ascribed to the mesoporosity of AC800. The isotherms reflected the Type IV isotherms of IUPAC classification for mesopore-containing materials [32]. The SBET of AC800 was 1058 m2/g. PSD and cumulative pore volume of AC800 obtained from the NLDFT method are indicated in Figure 2b. Table 1 gives the SBET and pore volume data. Both microporous (<2 nm) and mesoporous (2 nm–50 nm) structures can be observed in the PSD curve by the results of adsorption-desorption isotherms. The AC800 had a mesoporous dominant nature, and mesopores consisted of 65.85% of the total pore volume. This result showed that the waste textiles could use to produce AC with a porous network. A well-distributed porous network is essential for the mobility of the electrolyte ions throughout the pores. Better ion transport leads to lower charge transfer resistance and, consequently, better specific capacitance, energy density and power density [33]. The yield of the AC production process was 21.14%.

### 3.2. FTIR and XPS analysis

**Figure 3:**
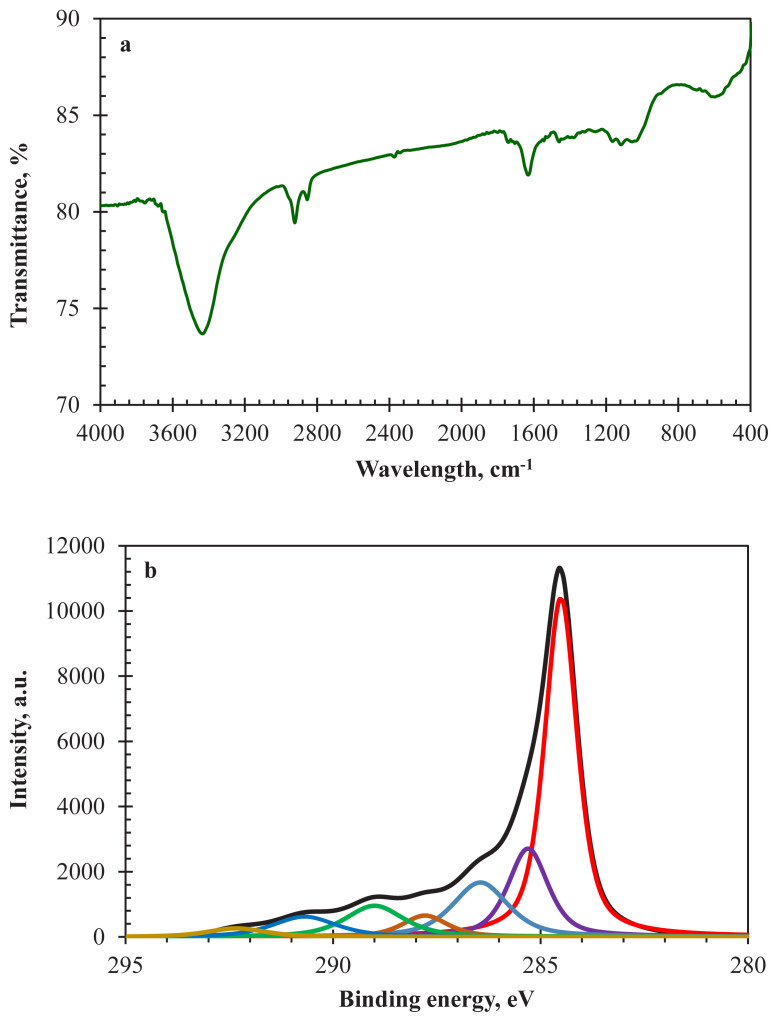
a) FTIR spectrum, b) XPS C1s spectrum and c) XPS N1s spectrum of AC800.

FTIR is an important characterisation technique to determine the type of organic surface functionalities on the surface of the AC sample. Figure 3a indicates the FTIR spectrum of the AC800. The absorption band detected between 3600–3300 cm^−1^ wavenumber region is ascribed to O — H stretching vibrations. AC samples may have different surface groups containing O — H bonds, such as phenolic hydroxyl, alcohol groups, and absorbed moisture. Aliphatic C — H stretching vibration is detected between 3000–2850 cm^−1^ wavenumber range. The C — O stretching vibrations of conjugated carbonyl groups showed at 1736 cm^−1^. This absorption band can be attributed to carboxylic acid, lactonic, and/or esteric groups [34]. The peak at 1674 cm^−1^ is related to nonconjugated carbonyls. The intense band at 1628 cm^−1^ can be assigned to highly conjugated carbonyls and C — C structure in aromatics. The band at 1528 cm^−1^ corresponding to vibrations of C — N stretching with N — H in-plane bending proves the existence of nitrogen-containing functional groups [35].

In addition to the FTIR spectrum, XPS C1s and N1s spectra of AC800 are given in Figures 3b and 3c to characterise the surface functional groups. Six subpeaks were obtained after the deconvolution of the C1s spectrum (Figure 3b). The peaks around 284.5 eV, 285.3 eV, 286.5 eV, 287.8 eV, 289.0 eV, and 290.7 eV - 292.4 eV correspond to sp^2^ hybridised carbons, sp^3^ hybridised carbons, C — O and C — N containing functional groups (hydroxyl, phenol, ether), carbonyl, C — O containing groups (carboxyl, ester, anhydride, lactone) and π — π* shake-up satellites, respectively. The subpeak compositions were 49.86%, 15.96%, 13.62%, 4.35%, 8.16%, 5.82%, and 2.23%, respectively. The existence of sp^2^ hybridised carbons is important for the electrical conductivity of AC800. Better electrical conductivity leads to lower resistance and better capacitive performance for the supercapacitor [36]. On the other hand, Figure 3c shows the deconvoluted peaks of the N1s spectrum. Four subpeaks were observed around 398.1 eV, 399.9 eV, 401.3 eV, and 402.4 eV with compositions of 4.21%, 52.63%, 30.65%, and 12.51%, respectively. These peaks are attributed to pyridinic-N, pyrrolic-N, graphitic-N, and oxidised-N groups, respectively. It is known that the pyridinic and pyrrolic-N functionalities are essential for the better pseudocapacitive performance of carbon-based electrode materials because of good electron donor contributions [7,37].

### 3.3. Electrochemical tests

#### 3.3.1. Three-electrode cell configuration

**Figure 4:**
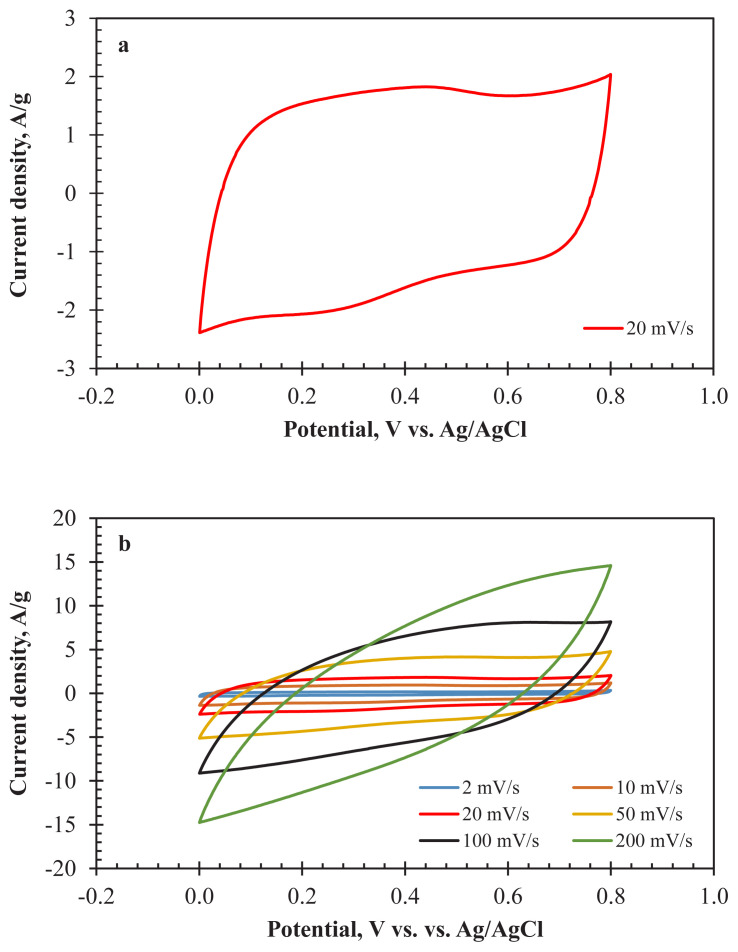
CV curves of AC800 a) at 20 mV/s, b) between 2–200 mV/s for three-electrode configuration (1 M H_2_SO_4_).

The usability of AC800 as an electrode material for supercapacitor applications was tested by the three-electrode system with 1 M H_2_SO_4_ as an aqueous electrolyte. Figure 4a shows the CV curves obtained for AC800 between 2–200 mV/s. A rectangular curve with small redox peaks was observed for a 20 mV/s scan rate. The rectangular shape of the curve reflected the double-layer capacitive property of AC800. On the other hand, the waste fabric samples contained 2% of elastane. Elastane is the term used in Europe for polyurethane fibres, and these kinds of synthetic fibres are used to increase the elasticity of fabrics [38]. Some of the nitrogen-containing groups were spontaneously involved in the structure of AC800 samples. The redox peaks (oxidation at 0.4 V and reduction at 0.25 V) observed originated from the nitrogen functional groups that existed on the surface of AC800 and introduced pseudocapacitive contribution. As a result of the increase in the scan rate from 2 mV/s to 200 mV/s, the rectangular shape of the curves disappeared, and no redox peaks were observed (Figure 4b). In other words, EDLC and pseudocapacitive behaviours of the electrode material decreased. This result was mainly due to the insufficient transportation of electrolyte ions to the pores of AC800. High scan rates affect the accessibility of pores and the pseudocapacitive character related to this property. Most of the heteroatoms in the structure of the activated carbons were located on the internal surfaces. Therefore, inadequate electrode diffusion and shorter retention time in the pores limited the possibility of the Faradaic reactions. As a result, the capacitive performance of AC800 decreased. Possible Faradaic reactions could be occurred due to the existence of oxygen and nitrogen-containing functional groups as follows [39–42]:


$$\begin{array}{l} -\text{C}-\text{OH}↔-\text{C}=\text{O}+{\text{H}}^{+}+{\text{e}}^{-}\\ -\text{COOH}↔-\text{COO}+{\text{H}}^{+}+{\text{e}}^{-} \end{array}$$

**Figure 5:**
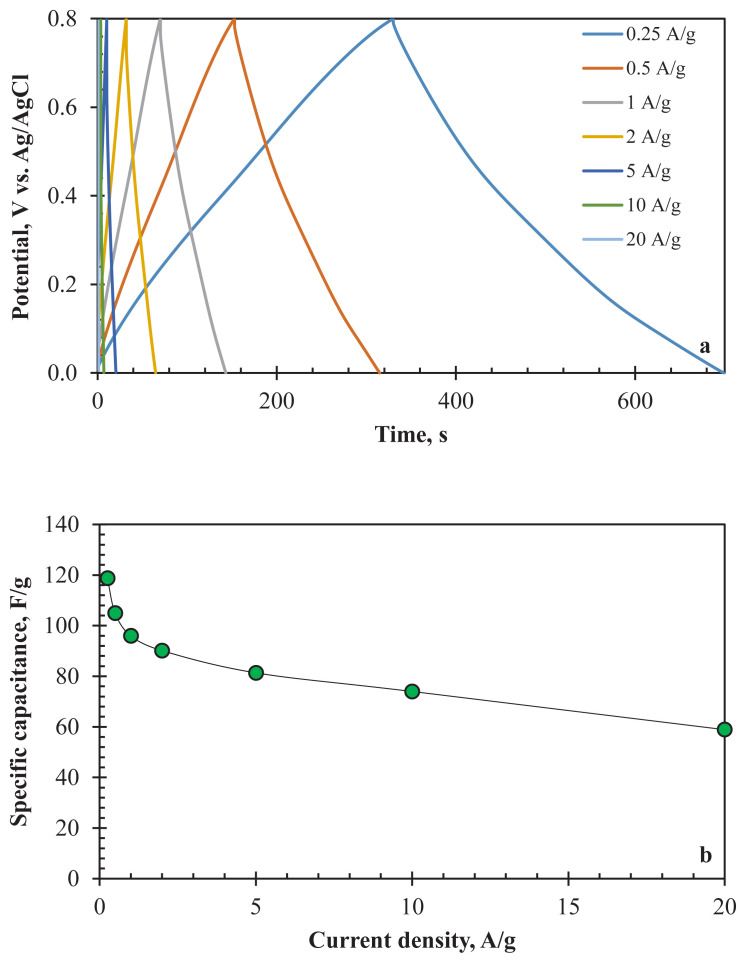
a) GCD curves, b) specific capacitance values (at 0.25–20 A/g), c) Nyquist plots, d) long-term stability (at 2 A/g for 1000 cycles) of AC800 for three-electrode electrochemical cell (1 M H_2_SO_4_).

The decreased pseudocapacitive effect was also observed in GCD analyses. Figure 5a depicts the GCD results of AC800 at various current density values for the three-electrode configuration. Depending on the faradaic reactions at 0.25 A/g and 0.5 A/g constant current densities, the slopes of the charge and discharge curves changed. The nonlinear curves at these current densities indicated the pseudocapacitive behaviour of the AC800 thanks to heteroatom content. The change in the slope of the curves refers to redox reactions at that voltage. However, the linearity of the curves increased with the change of current density values from 0.25 A/g to 20 A/g. Using high current density causes the loss in the pseudocapacitive behaviour of the electrode, and consequently, EDLC-type behaviour becomes dominant. As obtained in the CV analysis results, low ion diffusion through the pores at high current densities led to the minimisation or prevention of surface reactions. In addition, IR drops were observed from the GCD curves for all current densities. Higher IR drops with increasing current density (from 9.5 mV at 0.25 A/g to 327.8 mV at 10 A/g) correspond to higher energy losses.

Figure 5b shows the rate capability of the electrode between 0.25 A/g and 20 A/g current density values. The drop in specific capacitance value is visible in the figure. The specific capacitance was 118.73 F/g at 0.25 A/g, while it was only 58.89 F/g at 20 A/g. The disappearance of the pseudocapacitive property and increasing number of inaccessible pores of the electrode material caused a 54% reduction in specific capacitance. However, considering the 80-fold increase in current density, it can be said that the AC800 electrode has good rate capability.

Figure 5c depicts the typical Nyquist plot of the electrode. The semicircle observed corresponds to the charge transfer resistance of the electrode. This resistance is connected to the electrical conductivity and the porous structure of the electrode material. Inadequate pore size affects the electrolyte ion transportation through the pores and leads to poor electrochemical performance. Diffusion of electrolyte ions increases with well-distributed pore size, thereby reducing ionic resistance [43,44]. The large semicircle observed in the Nyquist plot represents limited electrical conductivity and pore distribution of AC800. However, the vertical line seen at low frequencies represents a nearly ideal capacitive property of the electrode. On the other hand, the intercept of the semicircle on the x-axis at high frequencies defines as equivalent series resistance (ESR). ESR can be defined as the total of the electrolyte resistance, the internal resistance of the electrode, the ohmic resistance of the current collector, and the contact resistance between the components in the cell configuration [45,46]. The ESR of the cell was determined as 0.45 Ω. EIS analysis was repeated after 1000 cycles of the GCD test to observe the change in charge transfer resistance. Almost the same trend in the Nyquist plot was obtained after 1000 cycles. The minor decrease in the diameter of the semicircle is probably due to the increasing wettability of the pore during the GCD analysis. This result indicated the long-term cyclic stability of AC800 in terms of charge transfer resistance.

The capacitance retention of AC800 for 1000 cycles was tested by GCD analysis at 2 A/g. Figure 5d shows the long-term stability of AC800. The initial specific capacitance was 89.93 F/g, while it was 88.73 F/g for the last cycle. The results show that the electrode maintains its capacitive performance to a great extent. Only a 1.33% reduction was detected after 1000 cycles. The high capacitive retention supports that AC800 can be used as electrode material for supercapacitors.

#### 3.3.2. Two-electrode cell configuration

**Figure 6:**
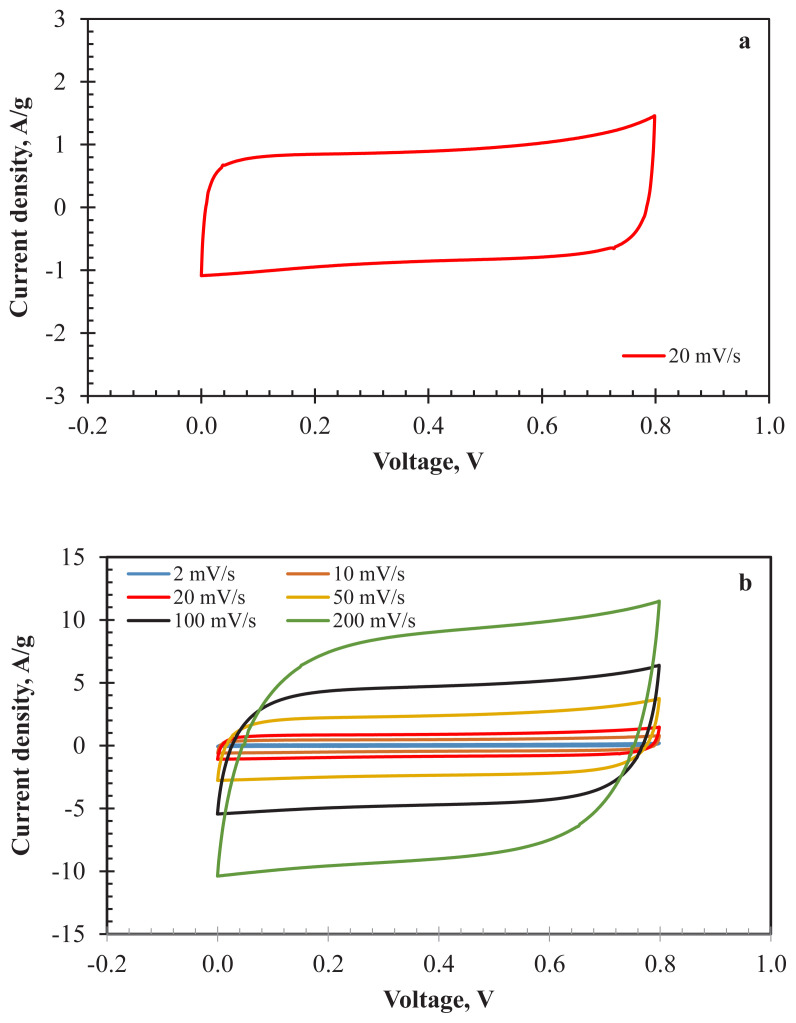
CV curves of AC800 a) at 20 mV/s, b) between 2–200 mV/s for two-electrode configuration (1 M H_2_SO_4_).

For the practical application of AC800, the two-electrode cell configuration was tested with 1 M H_2_SO_4_ electrolyte. The thicknesses of the electrodes were around 40 μm. Figure 6 indicates the CV analysis results of the prepared cell at various scan rates (2–200 mV/s). The rectangular curve at 20 mV/s (Figure 6a) reflects the ideal EDLC characteristic of the cell. The curves maintain their rectangular behaviour even at high scan rates (Figure 6b). Minor deviations detected at high scan rates corresponded to the fast charge-discharge transportation effect of the electrolyte ions. It can be reported that the PSD of the AC800 is sufficient enough for the diffusion of charged ions.

**Figure 7:**
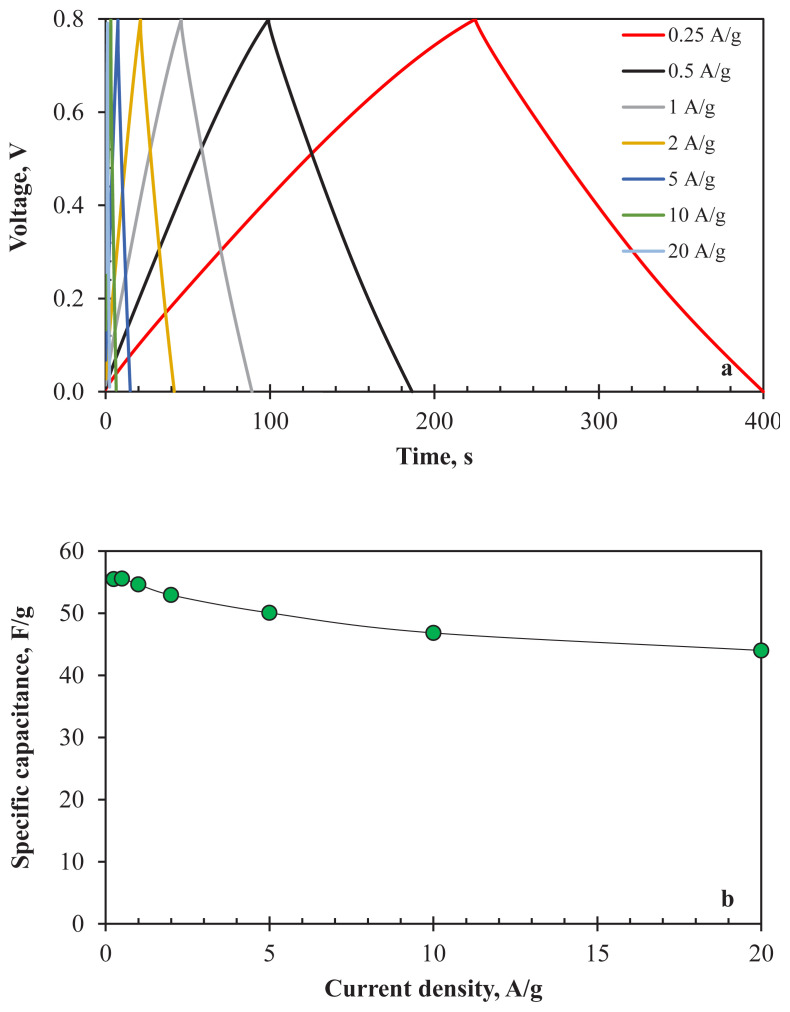
a) GCD curves, b) specific capacitance values (at 0.25–20 A/g), c) long-term stability (at 1 A/g for 3000 cycles), d) Nyquist plots of AC800 for two-electrode electrochemical cell (in 1 M H_2_SO_4_).

The electrochemical stability tests were conducted by GCD analyses. Figure 7a indicates the GCD curves of prepared supercapacitor cell between the current densities of 0.25 A/g and 20 A/g. The results exhibited a linear charge-discharge profile for all current densities. Lower IR drops (4.4 mV at 0.25 A/g and 221.2 mV at 10 A/g) were observed for the two-electrode configuration. Specific capacitance values of the cell obtained by applying several current density values are shown in Figure 7b. The cell shows acceptable capacitive performance for all current density values. The capacity loss between 0.25 A/g (55.48 F/g, 7.49 F/cm^3^) and 20 A/g (44.00 F/g, 5.94 F/m^3^) is only 20.7%. Significant chemical stability behaviour at high current densities is consistent with the CV analysis results.

The practical use of an electrode material for supercapacitor application depends on its long-term stability performance. The long-term electrochemical stability of AC800 was determined by applying 3000 GCD cycles at 1 A/g (Figure 7c). The capacity retention of the cell was determined as 83.01%, showing that AC800 is a potential electrode material candidate for supercapacitors. The decrease in electrochemical performance may be ascribed to the textural characteristics of the sample. It is thought that electrochemical performance can be increased by improving the surface area and pore structure of AC800. In addition, energy and power density values obtained from Equations 1 and 2 were 4.69 Wh/kg and 392.68 W/kg at 1 A/g.

Nyquist plots of the cell for the first and 3000th cycles are given in Figure 7d. It is clear from the figure that capacitive behaviour of the cell decreased after 3000 charge discharge cycles and consisted with the results in Figure 7c. The deviation from the vertical line corresponds to decreasing mobility of the electrolyte ions inside the cell, especially in the AC800 pores. ESR of the cell was also increased from 0.18 Ω to 0.32 Ω, supporting the increase in electrolyte resistance.

The results obtained were compared with similar studies in the literature (Table 2). The table only includes studies on activated carbons produced from textile products. There are limited number of studies in current literature. Different activating agents were used in these studies. The structural and chemical characteristics of the samples varied according to the activation chemical and production method. All the results are quite acceptable and clearly show that textile wastes can be used as electrode materials for supercapacitors. The activated carbon produced in this study has the second-highest specific capacitance value among the electrode materials. Melamine modified activated carbon showed by far the best performance. However, it should be noted that this sample involves additional production steps.

## 4. Conclusions

Textiles are recognised as worldwide wastes that have the potential to be recycled into valuable products after their life cycle. The waste textile product (denim) was selected as a starting material in this study to produce activated carbon (AC800). Considering to porous nature and the heteroatom composition of the produced activated carbon, its electrochemical performance was tested in a supercapacitor cell. The electrochemical performance tests proved that the waste jean could be used as a suitable electrode material for supercapacitors. The three-electrode cell configuration results showed the pseudocapacitive behaviour of AC800 due to its heteroatom content. The cell showed good cyclic stability (1.33% decrease) after 1000 cycles for three-electrode system. Gravimetric and volumetric specific capacitance values obtained by three-electrode and two-electrode electrochemical cell configurations were 95.93 F/g (12.95 F/cm^3^) and 54.64 F/g (7.38 F/cm^3^) at 1 A/g. The results are promising, and it is thought that electrochemical characteristics can be increased by improving the textural properties of AC800. In particular, a more convenient diffusion route can be prepared for the electrolyte ions by increasing the pore volume in the 1–6 nm region.

## Figures and Tables

**Table 1:** Textural properties of AC800.

BET surface area, m^2^/g	Total pore volume, cm^3^/g	V_micro_^a^, cm^3^/g	V_meso_^b^, cm^3^/g	V_mic_, %	V_meso_, %
1058	0.8526	0.2997	0.5529	35.15	65.85

a micropore volume (at 2 nm),

b mesopore volume (>2 nm, NLDFT).

**Table 2:** Comparison of electrochemical performances of waste textile-based supercapacitors for two-electrode configuration.

Material	Activating agent	BET surface area, m^2^/g	Specific capacitance, F/g	Reference
Recycled jute	KOH	1769	51 (at 5 mV/s)	[27]
Denim fabric	H_3_PO_4_	2032	35 (at 1 A/g)	[28]
Melamine modified denim fabric	LiCl-KCl	1975.2	130 (at 1 A/g)	[30]
MnO_2_ deposited cotton t-shirt	NaF	Not specified	51 (at 5 mV/s)	[47]
Denim fabric	KOH	1058	54.64 (at 1A/g)	This work
